# Supplementary material for: When animals cry: The effect of adding tears to animal expressions on human judgment
Source: PLoS One. 2021 May 6;16(5):e0251083. doi: 10.1371/journal.pone.0251083 (PMC8101718; doi:10.1371/journal.pone.0251083)
Supplement: Attachment — Submitted filename: Response to reviewers (2nd).docx [file pone.0251083.s002.docx]

Journal Requirements:

Please **review your reference list** to ensure that it is complete and correct. If you have cited papers that have been retracted, please include the rationale for doing so in the manuscript text, or remove these references and replace them with relevant current references. Any changes to the reference list should be mentioned in the rebuttal letter that accompanies your revised manuscript. If you need to cite a retracted article, indicate the article’s retracted status in the References list and also include a citation and full reference for the retraction notice.

**RESPONSE:** The reference list is revised. No retracted papers. A reference was changed after reading a comment from the reviewer (please check the response below)

Comments to the Author

**Reviewer #1:** Overall, I would like to thank the authors for having addressed my previous points so thoroughly! Taking this into account, I find the present version of this manuscript to be substantially improved. In particular, I appreciated the inclusion of the additional post-test and analyses included in the supplementary materials that, I think, are very helpful with respect to better understanding the strengths and limitations of the present work. Likewise, I would like to thank the authors for having performed additional fixed and random effects analyses. The finding that these results converged with the previous ANOVA-results suggest that these were indeed robust. It also makes sense to me to stick with presenting the simpler ANOVA results in this case.

As illustrated by the post-test, a few limitations still remain: the limited number of exemplars; the lack of realism for a few of the stimuli (horse, cat); the variability in the extent to which some of the tearless stimuli were perceived as emotional (e.g., horse vs. dog). I therefore still find some parts of the discussion regarding differences between animal species (e.g., between cats and dogs) to perhaps be a bit premature at this point. Nevertheless, it is of course interesting to speculate about such differences, and this is being done more cautiously now (and in view of the limitations of this initial study). The discussion has furthermore been substantially improved overall, and the main findings are presented more clearly now.

**RESPONSE:** Regarding the dog, we are in agreement with you in that it was seen as emotional even in the original tearless image, which we described as «unique » in the discussion, given the amount of emotionality rated on it. At this regard, we think it could be because a variety of reasons, including our relationship with dogs in general (as especulated in the Discussion) or also due to the chosen breed for the photography, or even to the expression of this concrete dog/photo. In any case, this illustrates the need for a careful selection of the images, and also the need of a quality study like the one we performed, following your suggestion. Please check a couple of new phrases included in the dog&cat argument as advices for caution with interpretations, and also a new phrase in the « Strenghts and limitations » section, in line with your comments.

Results of the new rating study:

I believe that the new post-test has helped to sufficiently strengthen this already very interesting paper. Since these materials are openly available, it should be easy to replicate/extend the current work. Tears were overall judged as very well visible in the new rating study. This, together with being able to view all the pictures in full helps to address a lot of the concerns about the technical soundness of this work. However, as shown by the study of the stimuli, some limitations do remain. First, perceived emotionality of the tearless images appears to have been quite variable. In particular, the tearless horse picture seems to have been perceived as substantially less emotional than the dog picture (which was already perceived as very emotional even without tears). This suggests that some of the effects of tears on specific animal images may indeed be more difficult, and perhaps more interesting, to interpret (see discussion). Second, it seems that the tears on the Horse and Cat may not have been perceived as sufficiently realistic. I.e., here, it would seem that the answer to this question from the response letter: "provided they were perceived as realistic from the new study" - may have to be that this is not fully supported by the post-test. Nevertheless, neither of these limitations would seem to invalidate/threaten the conclusion that the addition of tears generally increased perceived emotionality.

**RESPONSE:** The phrase you cite -copied from the rebuttal letter- is certainly not fully supported by the post-test (surely due to an excess of abstract for the letter), and in fact it was not included in the text of the manuscript. Instead, in the anterior manuscript it can be read: *“Finally, we noted a few unexpected results (the “colder tearful cat” and the absence of a tear effect on aggressiveness in the horse) that were obtained with the pictures that were rated as less realistic in the quality study. This prompts an interesting reflection on the use of artificial stimuli to depict human emotions in non-human targets (animals and possibly robots), in the sense that, the more artificial and unreal they are perceived to be, the more difficult it is for them to produce empathy in us, and thus an anthropomorphic feeling”* We think that this paragraph is expressing the limitations you note in a constructive manner.

I only have a few minor additional comments beyond the above-mentioned limitations of the post-test:

Introduction:

I was not sure if quite as much background on human eye gaze is needed here. The first paragraph might be shortened somewhat to move more quickly towards tears, or animal crying.

**RESPONSE**: The first paragraph has been adequately shortened.

Regarding reference [9], i.e., responses to tears within 50 milliseconds - here, recent work has so far failed to replicate this finding (Gračanin et al., 2021).

Reference:

Gračanin, A., Krahmer, E., Balsters, M., Küster, D., & Vingerhoets, A. J. (2021). How weeping influences the perception of facial expressions: The signal value of tears. Journal of Nonverbal Behavior, 45(1), 83-105.

**RESPONSE:** We thank you for this reference, which we have read with great interest. After carefully reading, we have decided to change a bit our sentence and to eliminate the commented reference (number 9: Balsters et al., 2013). We noted that both the Balsters paper and the attempt to replicate its findings by Gračanin et al. assess reaction times and not the influence of a preattentional stimulus. Given that our aim was not the measurement of exact reaction times, and that this paragraph of the Introduction was written just to generally point that we (and our brain) in fact react to emotional tears, we now cite a reference we found in the literature on bioelectrical changes caused by the sight of tears: Krivan S, Caltabiano N, Cottrell D, Thomas N. I'll cry instead: Mu suppression responses to tearful facial expressions. Neuropsychologia. 2020;143:107490.

Minor points:

p.5, l.134: there is an opening "(" here that is missing the closing ")".

p.5, l.154: replace "according their lacking of..." with "according to their lack of..."

p.6, l.174: replace "for such process" with "for such [a] process"

p.16, l.450: replace "confirmed our hypothesis" with "supported our hypothesis"

**RESPONSE**: All these minor points have been observed and corrected.
